# Adverse birth outcomes and its associated factors among women who delivered in North Wollo zone, northeast Ethiopia: a facility based cross-sectional study

**DOI:** 10.1186/s13104-019-4387-9

**Published:** 2019-06-24

**Authors:** Eskeziaw Abebe Kassahun, Habtamu Dessie Mitku, Mikiyas Amare Getu

**Affiliations:** 1 Department of Midwifery, Faculty of Health Sciences, Woldia University, Woldia, Ethiopia; 2 Department of Statistics, Faculty of Natural and Computational Sciences, Woldia University, Woldia, Ethiopia; 3 Department of Nursing, Faculty of Health Sciences, Woldia University, Woldia, Ethiopia

**Keywords:** Adverse birth outcomes, Preterm birth, Stillbirth, Low birth weight, North Wollo zone, Ethiopia

## Abstract

**Objective:**

Pregnancy is a happy time for women and their families although it can also a period of concern and anxiety, for some women, lose their babies during early or late pregnancy, give birth earlier than the expected date or face low birth weight. Therefore, this study aimed to assess the prevalence of adverse birth outcomes and associated factors among women who delivered in the North Wollo zone, northeast Ethiopia.

**Result:**

The prevalence of adverse birth outcomes among women who delivered in North Wollo zone was 31. 8%. Living in rural area (AOR = 1.8; 95% CI 1.13, 2.78), age ≥ 34 years (AOR = 2.2; 95% CI 1.21, 4.05), mid-upper-arm circumference < 23 cm (AOR = 3.1; 95% CI 1.90, 4.94), multigravida women (AOR = 1.8; 95% CI 1.08, 3.06), lack of antenatal care visit (AOR = 2.1; 95% CI 1.02, 4.40) and complications during pregnancy (AOR = 2.1; 95% CI 1.23, 3.55) were significantly associated with adverse birth outcomes. The prevalence of adverse birth outcomes was high and is a major public health problem in Ethiopia particularly in the study area. Hence, increasing the access to health institutions, developing strategies to prevent and treat complications, and providing focused antenatal care follow-up for pregnant women is recommended.

## Introduction

Pregnancy is usually a happy time for most women and their families although it can also be a period of concern and anxiety, for some women lose their babies during early or late pregnancy and delivery earlier than the expected date or face low birth weight [1].

Adverse birth outcomes are common health problems in developing countries [2] and incur significant health consequences on infants, and emotional and economic costs on families, and communities across the world, particularly in resource-limited settings where health systems and access to and utilization of health services are weak [3–5].

Globally, preterm birth is the leading cause of neonatal deaths which is contributing to 35% of the world’s neonatal mortality. It’s also the leading cause of disability and ill health later in life. Out of 7.6 million deaths of under-five children, 17% are due to prematurity [6]. Each year, 15 million babies are preterm birth and more than one million dies [7]. As a result, many of the survivors face physical and neurological problems and, the onset of communicable and non-communicable diseases or educational disabilities [7, 8] for life. More than 60% of the preterm births take place in South Asia and Sub-Saharan Africa [7]. According to the Ethiopian profile of preterm and low-birth weight prevention and care report, 320,000 babies are born in each year of which 24,400 die at the age of less than 5 years due to direct complications of preterm birth [9].

Low-birth-weight babies experience growth retardation and inhibited cognitive developments, besides, to severe health problems from immediately after birth to later life [10, 11].

Globally, more than 20 million infants (15.5% of all births) are born with low birth weight, 95.6% of them in developing countries. Around 15% of low-birth-weight occurs in Sub-Saharan Africa [12]. According to the 2016 Ethiopian Demographic and Health Survey (EDHS) report, 13% of infants weighed less than 2.5 kg at birth [13].

Worldwide, over 2.6 million deliveries are stillbirths 98% of which occur in low and middle-income countries. About 60% of the stillbirths are in rural areas, and more than half occur among conflict and emergency zones [14, 15].

Although the Ethiopian government has developed strategy and plan to improve the wellbeing of mothers and children [16], the level of adverse birth outcomes are a major public health problem in areas such as Shire, Tigray, (22.6%) [17], Gondar (23%) [18], and Hosanna, (24.5%) [19]. Consequently, the mortality rate of neonates, infants, and under five-children has been 29, 48, and 67 per 1000 live births, respectively [13]. Therefore, this study aimed to assess the prevalence of adverse birth outcomes and associated factors among women who delivered in the health institutions of North Wollo.

## Main text

### Methods

#### Study design and settings

An institution based cross-sectional study was conducted on 462 delivered mothers from March 2017 to June 2018. North Wollo zone is found 521 km from Addis Ababa, the capital of Ethiopia. It has 65 health centers and 3 hospitals. According to the 2017 Zonal Health Administrative Office report, 65 health centers gave delivery services to 600 women, while the three hospitals served 60 women per week. About 24% of the women were of childbearing age.

#### Sample size and sampling procedures

All women who delivered in North Wollo zone health institutions were included in the study. However, women who gave birth with unknown last normal menstrual period (LNMP) or who had no a reliable ultrasonography results and severely ill were excluded. The required sample size was calculated using the single population proportion formula by considering the proportion of adverse birth outcomes as 22.7% taken from a previous study in Gondar [20], a 95% confidence interval (CI), 4% margin of error (d) and 10% non-response rate; the final sample was 462 women. First, the health institutions were stratified into hospitals and health centers. Then, by the random sampling method and rule of thumb (20%), thirteen health centers and one hospital were selected. Finally, using the systematic random sampling method and proportional allocation to each selected health institution, 462 women were selected and interviewed.

#### Measurement

##### Adverse birth outcomes

A baby is said to be preterm, if born before 37 completed weeks of gestation but after 28 weeks of gestation or low birth weight, if its birth weight is below 2500 g and stillbirth, if the infant died in the womb or during intrapartum period after 28 weeks of gestation [21]. If a woman met at least one of the above conditions, it is considered as an adverse birth outcome.

### Data collection tools and techniques

Data was collected through pretested and structured interviewer-administered questionnaire. It was first prepared in English and translated to Amharic (the local language), and back-translated to English again. A total of 28 data collectors (BSc. in Midwifery) were involved in the data collection process. The purposes and objectives of the study were clearly explained to participants before data collection.

#### Data quality controls

The questionnaire was pretested on 24 women in Kobo primary hospital out of the study area. Twenty-eight data collectors were selected from the selected health institutions. Interviewers received a total of 15 days of intensive training before data collection. During data collection, any personal identifiers were not recorded.

#### Data processing and analysis

After the data collection, data was entered in EpiData 3.10 and exported to SPSS version 20 for analysis. Both bivariable and multivariable logistic regression analysis were carried out to identify factors associated with adverse birth outcomes. In bivariate logistic regression analysis, variables with p-value less than 20% were considered into the multivariable analysis to control the possible effect of confounders. Adjusted odds ratio (AOR) with a 95% confidence interval (CI) was calculated to see the strength and significant association. Variables having a p-value less than 0.05 in the multivariable logistic regression analysis were considered as statistically significant.

### Results

#### Sociodemographic and economic characteristics

A total of 462 women were interviewed, which makes a response rate of 100%. The majority (76.8%) of the women were in the age range of 20–34 years. About 61.9 and 93.5% of women lived in urban areas and married, respectively. Moreover, 26.4% of women were illiterate and 38.7% were farmers (Table 1).

**Table 1:** Sociodemographic, medical and behavioral characteristics of respondents in North Wollo zone, northeast Ethiopia, 2017 (n = 462)

Variables	Frequency	Percentage
Religion		
Orthodox Christian	385	83.3
Protestant	3	0.6
Muslim	74	16.1
Age (years)		
< 20	39	8.4
20–34	355	76.8
> 34	68	14.7
Family size		
≤ 5	403	87.2
> 5	59	12.8
Marital status		
Married	432	93.5
Unmarried	30	6.5
Women’s education		
Unable to read and write	122	26.4
Able to read and write	81	17.5
Primary (1–8th) school	87	18.8
Secondary (9–12th) school	72	15.6
College or higher education	100	21.6
Partner’s occupation		
Governmental employee	129	27.9
Merchant	96	20.8
Farmer	179	38.7
Private employer	34	7.4
Others^a^	24	5.2
Time to reach nearby health institution (min)		
≤ 30	223	48.3
> 30	239	57.7
Maternal MUAC (cm)		
< 23	131	28.4
≥ 23	331	71.6
History of poor pregnancy outcomes		
No	54	11.7
Yes	408	88.3
Complication during pregnancy		
Yes	83	18.0
No	379	82.0
Type of complication during pregnancy (n = 83)		
APH	18	21.7
PROM	12	14.5
PIH	25	30.1
Others^b^	28	33.7

^a^Daily laborer, jobless, driver or soldier: ^b^ nausea and vomiting, infections, trauma

#### Obstetrics characteristics

The majority (91%) of women had an ANC follow-up. Of those, 20.7, 69.3 and 10% of women started ANC visit during the first, second and third trimester of pregnancy, respectively (Table 2).

**Table 2:** Obstetrics characteristics of women delivered in North Wollo zone health institutions, northeast Ethiopia, 2017 (n = 462)

Variables	Frequency	Percentage
ANC follow up		
Yes	420	90.9
No	42	9.1
Frequency of ANC follow up (n = 420)		
< 4 times	168	40.0
4 times	189	45.0
> 4 times	63	15.0
First ANC visit (n = 420)		
First trimester	87	20.7
Second trimester	291	69.3
Third trimester	42	10.0
Iron/folic acid intake		
Yes	389	84.2
No	73	15.8
Maternal gravidity		
Primigravida	184	39.8
Multigravida	278	60.2
Labor status		
Spontaneous	442	95.7
Induced	20	4.3

### Prevalence of adverse birth outcomes

The overall prevalence of adverse birth outcomes was 31. 8% (95% CI 27.6%, 36.1%). Of those, one-fifth (19.5%) were low birth weight, while 13.2% were preterm and 7.8% stillbirths.

### Factors contributing to adverse birth outcomes

Both bivariable and multivariable logistic regression analyses were done to see the effects of the selected variables on adverse birth outcomes. As it is shown in Table 3, residence, age, mother’s education, MUAC, gravidity, ANC visits and complications during pregnancy had significant associations with adverse birth outcomes in the bivariable analysis. However, in the multivariable logistic regression analysis residence, age, MUAC, gravidity, ANC visit and complications during pregnancy were significantly and independently associated with adverse birth outcomes. Accordingly, the odds of having adverse birth outcomes increased by 80% (AOR = 1.8; 95% CI 1.13, 2.78) among women who lived in a rural area as compared with women who lived in an urban area. The likelihood of encountering adverse birth outcomes was 2.2 times (AOR = 2.2; 95% CI 1.21, 4.05) higher among women aged ≥ 34 years compared to women aged 20–34 years. Moreover, the odds of developing adverse birth outcomes among women whose MUAC was less than 23 cm were 3 times (AOR = 3.1; 95% CI 1.90, 4.94) higher compared to women whose MUAC was ≥ 23 cm.

**Table 3:** Bivariable and multivariable analyses of adverse birth outcomes of women delivered in North Wollo zone health institutions, northeast Ethiopia, 2017

Variables	Adverse birth outcomes		COR (95%)	AOR (95%)
	Yes	No		
Residence				
Urban	75	211	1.0	1.0
Rural	72	104	2.0 (1.31–2.91)	1.8 (1.13, 2.78)*
Age (years)				
< 20	8	31	0.6 (0.27–1.34)	0.5 (0.20, 1.20)
20–34	107	248	1.00	1.00
≥ 34	32	36	2.1 (1.22–3.49)	2.2 (1.21, 4.05)*
Mother’s education				
Unable to write and read	50	72	2.6 (1.43, 4.77)	1.8 (0.90, 3.40)
Able to write and read	31	50	2.3 (1.20, 4.50)	1.4 (0.65, 2.83)
Primary school	23	64	1.4 (0.69, 2.66)	1.6 (0.79, 3.42)
Secondary school	22	50	1.7 (0.83, 3.32)	2.0 (0.92, 4.94)
College and above	21	79	1.0	1.0
Maternal MUAC				
< 23	59	72	2.3 (1.48, 3.45)	3.1 (1.90, 4.94)*
≥ 23	88	243	1.00	1.00
Gravidity				
Primigravida	44	140	1.0	1.0
Multigravida	103	175	1.9 (1.23–2.84)	1.8 (1.08, 3.06)*
ANC visit				
Yes	126	294	1.0	1.0
No	21	21	2.3 (1.23, 4.42)	2.1 (1.02, 4.40)*
Complication during pregnancy				
Yes	39	44	2.2 (1.37, 3.61)	2.1 (1.23, 3.55)*
No	108	271	1.0	1.0

*Significantly associated factors at a p-value < 0.05

In this study, the higher odds of developing adverse birth outcomes were also observed among multigravida (AOR = 1.8; 95% CI 1.08, 3.06) than primigravida women. Likewise, the odds of exhibited adverse birth outcomes among women with complications during pregnancy were 3 times (AOR = 3.1, 95% CI 1.90, 4.94**)** higher compared to those of their counterparts (Table 3).

## Discussion

This study was conducted to assess the prevalence and factors associated with adverse birth outcomes among women who delivered in North Wollo zone health institutions, northeast Ethiopia. The overall prevalence of adverse birth outcomes was found to be 31.8% (95% CI 27.6%, 36.1%). This finding is higher than those of studies conducted in Gondar (23%) [18], Hosanna Town (24.5%) [19], Suhul Hospital, Tigray (22.6%) [17], Tanzania (18%) [22], and China (23.5%) [23]. The possible reason for the difference might be variations in the areas of studies. For instance, the studies in Gondar, Hosanna and Suhul hospitals were hospital-based unlike this study which was also conducted at health centers. Commonly, health centers are located in a rural area to provide services to rural women who are usually unemployed, overworked and have poor access to antenatal care, labor, and delivery services. Furthermore, methodological [24] and socio-economic [24, 25] variations may explain the differences in adverse birth outcomes in Ethiopia on one hand and Tanzania, Ghana and China on the other hand.

Women who live in rural areas are unemployed and less informed about pregnancy, labor and delivery. Moreover, cultural or traditional taboos have a great effect on the nutritional status of women through the prohibition of essential foods and or drinks, are commonly practiced in rural areas, such women are more likely to develop adverse birth outcomes than urban dwellers. Our finding is similar to those of studies reported in Gamo Gofa [26], China [23] and Hosana town [19].

In this study, women ≥ 34 years of age were at higher risk of encountering adverse birth outcomes. This is supported by other findings in China [23]. Perhaps because of the advanced maternal age result in adverse pregnancy outcomes [27, 28].

Women with a Mid Upper Arm Circumference (MUAC) less 23 cm were three times more likely to have adverse birth outcomes than women with MUAC ≥ 23 cm. This result is in agreement with those of other studies in Dessie [29]. That is because MUAC less than 23 cm might be related to maternal undernutrition which affects the fetus in the womb. Poor nutrition during child and adulthood might persist in the offspring, too.

Multigravida women were twice as likely to develop adverse birth outcomes than primigravida women. This finding is supported by other studies in Tigray [17] and Gamo Gofa [26]. This might be due to the socioeconomic burden which increases the sharing of food among family members.

Women who did not attend ANC were more likely to have adverse birth outcomes when compared to those who used the service. This finding is in line with those of studies in Dessie [29], Gondar [18] and Tanzania [22]. This might be because attending antenatal care helps women to have an awareness of the danger signs during pregnancy, delivery and postnatal period. It also improves health-seeking behavior, orients women on potential complications, birth readiness and help them to identify pregnancy-related problems.

Unrecognized and untreated pregnancy-related health problems put women and their fetus at risk of many problems. In this study, women with complications during pregnancy were more likely to have adverse birth outcomes. Again, this study is consistent with other studies in Dessie [29] and Hosanna town [19].

## Conclusion

This study revealed that the prevalence of the adverse birth outcomes in North Wollo zone was 31.8%. Residence, age, gravidity, Mid Upper Arm Circumference (MUAC), antenatal care (ANC) and pregnancy complications were mainly associated with adverse birth outcomes. Therefore, increasing the accessibility of health institutions, developing strategies and policies to prevent, diagnose and treat complications before and during pregnancy are recommended. Creating and raising awareness of women on the effect of pregnancy at an advanced age, and providing timely and focused antenatal care (ANC) follow up to all pregnant women are very important to minimize the problem.

## Limitations of the study

Being a cross-sectional study design attempt to our work did not establish the possible temporal relationship between dependent and independent variables. Besides, recall bias in finding out the gestational age of pregnant women was not ruled out.

## Data Availability

Due to ethical restrictions and privacy concerns, a dataset is available upon request from the author Eskeziaw Abebe: eskeziaw02@gmail.com.

## Abbreviations

ANC: antenatal care
CI: confidence interval
IQR: inter quartile range
MUAC: mid upper arm circumference
PIH: pregnancy induced hypertension
PPH: post-partum hemorrhage
PROM: premature rapture of membrane
SPSS: Statistical Package for Social Sciences
